# Thoracoscopic esophagectomy in the prone position for esophageal cancer patients with pectus excavatum: a report of two cases

**DOI:** 10.1186/s40792-021-01193-9

**Published:** 2021-05-07

**Authors:** Tomoya Tsukada, Yuto Kitano, Yuya Sugimoto, Masahide Kaji

**Affiliations:** Department of Surgery, Toyama Prefectural Central Hospital, 2-78 Nishi Nagae, Toyama, 930-8550 Japan

**Keywords:** Esophageal cancer, Pectus excavatum, Thoracoscopic esophagectomy, Case report

## Abstract

**Background:**

Pectus excavatum is a common thoracic deformity that can be encountered during thoracoscopic esophagectomy. Here, we report two cases of esophageal cancer complicated by pectus excavatum that were treated with thoracoscopic esophagectomy with the patients in the prone position.

**Case presentation:**

The first patient was a 64-year-old male diagnosed with esophageal cancer (cT3N0M0, Haller index 8.5) and underwent radical thoracoscopic esophagectomy in the prone position following neoadjuvant chemotherapy. The second patient was a 67-year-old male diagnosed with esophageal cancer (cT1bN0M0, Haller index 4.3), and the same procedure was performed in this patient. In cases of patients with a high Haller index, where securing the surgical field is difficult, preoperative computed tomography in the prone position can help surgeons to understand the mediastinal field of view and is safe.

**Conclusions:**

Radical thoracoscopic esophagectomy in the prone position may be a surgical option in patients with pectus excavatum.

**Supplementary Information:**

The online version contains supplementary material available at 10.1186/s40792-021-01193-9.

## Background

According to a nationwide survey by the Japanese association for thoracic surgery in 2017, more than 60% of esophagectomies in Japan have been performed thoracoscopically and/or laparoscopically [1]. Pectus excavatum (PE) is the most common thoracic deformity, with a prevalence of 0.1–0.2% in the general population [2]. Thus, it can be potentially encountered during esophagectomy. When we encounter such complicated case, the narrow working space and operative visual field in the mediastinum makes it difficult to perform intrathoracic surgery. There have been only a few reports on thoracoscopic esophagectomy in patients with PE, which was performed in the left lateral decubitus position [3, 4]. Herein, we report the first two cases of esophageal cancer complicated by PE that were treated with thoracoscopic esophagectomy in the prone position.

## Case presentation

### Patient 1

**Fig. 1:**
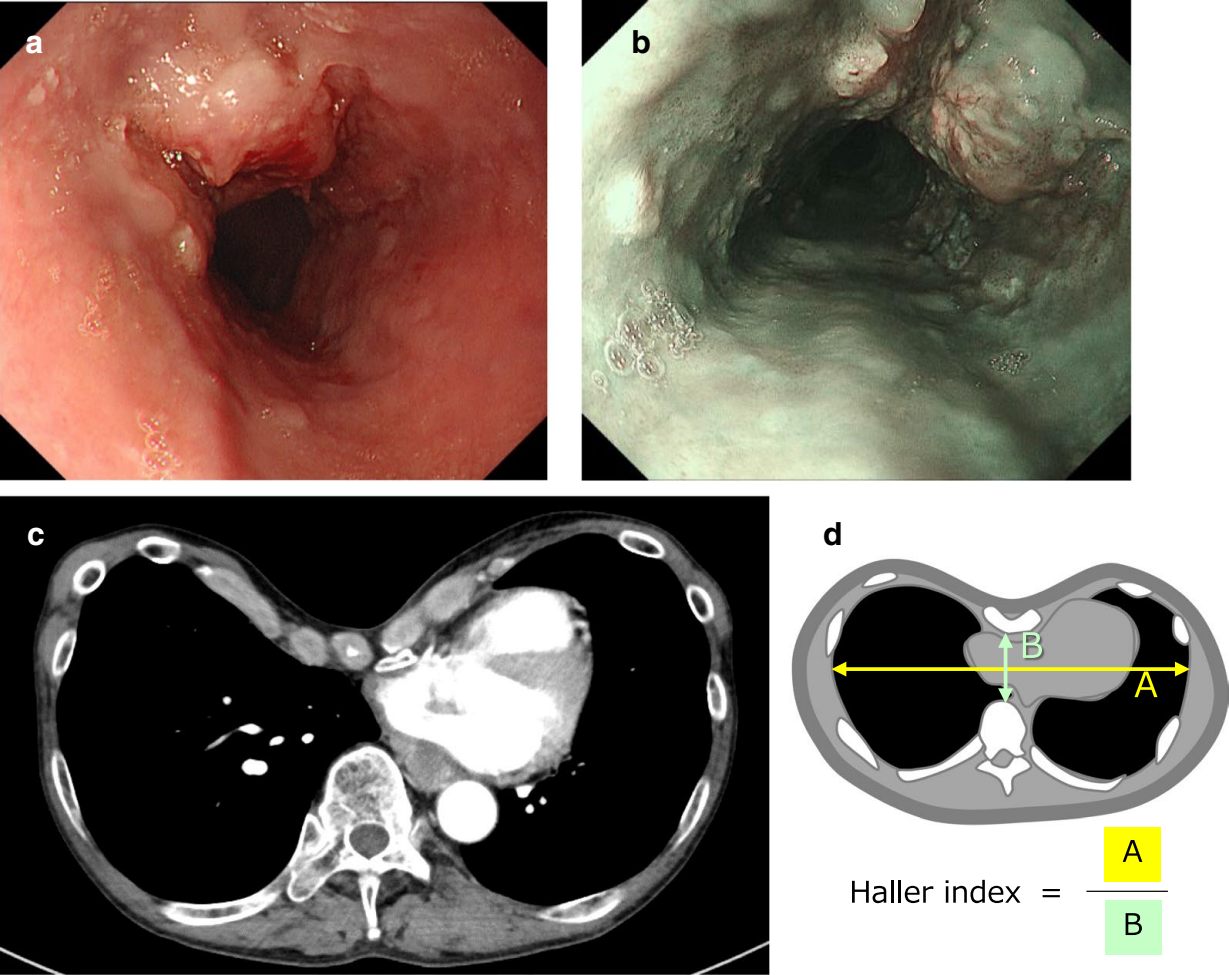
Patient 1. **a** Endoscopic findings. An elevated tumor with flat mucosal erosion was detected 33–38 cm from the upper incisors and suspected muscle invasion. **b** Narrow band imaging. **c** Contrast-enhanced CT reveals a hypovascular mass lesion in the middle esophagus and pectus excavatum. The Haller index score was 8.5. **d** Schema of the Haller index

**Fig. 2:**
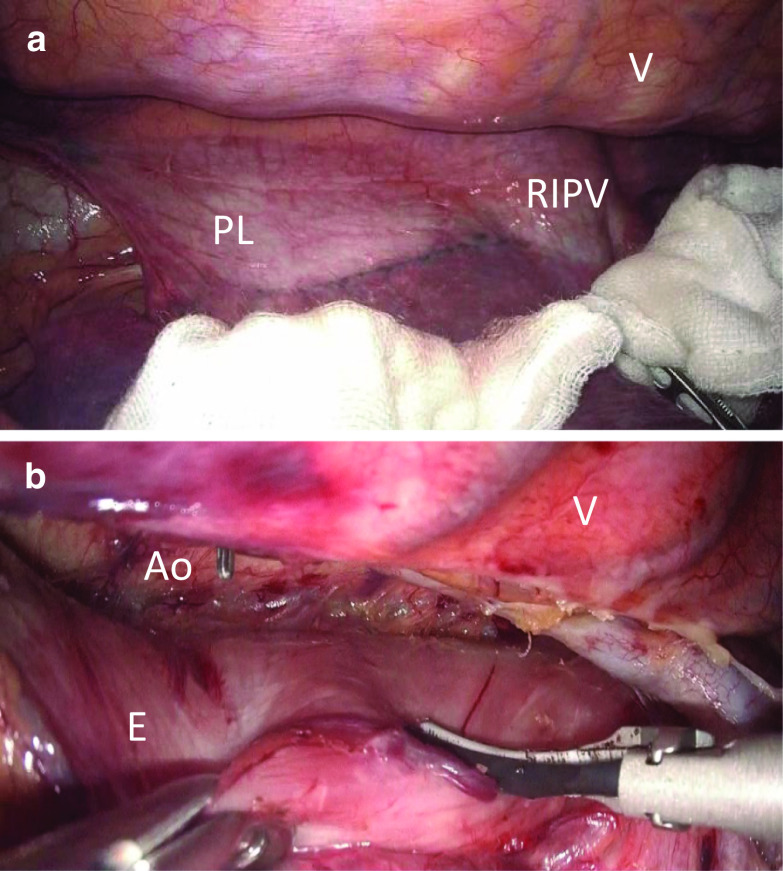
Thoracoscopic intraoperative findings of Patient 1. **a** Top-down visual field at the start of surgery. **b** Bottom-up visual field and the posterior mediastinum was found to be narrow because of pectus excavatum. *V* Vertebra, *PL* pulmonary ligament, *RIPV* right inferior pulmonary vein, *E* esophagus, *Ao* aorta

A 64-year-old Asian male was diagnosed with esophageal cancer during a routine medical checkup and was referred to our hospital for further treatment. Endoscopy revealed an elevated tumor with a flat lesion, 33–38 cm from the upper incisors (Fig. 1a, b); a biopsy revealed that it was a squamous cell carcinoma. Upon physical examination, the patient was found to have a significant PE. Contrast-enhanced computed tomography (CT) revealed a hypovascular lesion in the middle esophagus and PE (Fig. 1c). The Haller index (Fig. 1d), which is defined as the distance of the inner rib cage divided by the distance between the sternal notch and the vertebrae [5], was 8.5. The final preoperative diagnosis was a cT3N0M0 stage II squamous cell carcinoma of the esophagus, according to the Union for International Cancer Control (UICC) TNM classification (version 8) (used for all subsequent cancer classifications). Two courses of fluorouracil (800 mg/m^2^, 24 h continuous intravenous infusion on days 1–5), cisplatin (80 mg/m^2^, 2 h intravenous infusion on day 1), and docetaxel (30 mg/m^2^, 1 h intravenous infusion on days 1 and 15) were administered as neoadjuvant chemotherapy which is more intensive regimen [6, 7], instead of the Japanese standard neoadjuvant regimen (fluorouracil and cisplatin). Regarding PE treatment, the patient had no clinical symptoms, such as chest pain or dyspnea; therefore, only esophagectomy was performed after a respiratory surgeon was consulted. Radical thoracoscopic esophagectomy with three-field lymph node dissection was performed on the patient in the prone position. The patient was placed in the left semiprone position during the procedure. All procedures were performed in the prone position by rotating the surgical table. Three 12-mm ports were inserted into the fifth and seventh intercostal spaces on the posterior axillary line and the ninth intercostal space on the scapular angle line, and two 5-mm ports were inserted in the third and eighth intercostal space behind the midaxillary line. The endoscope was usually inserted through the ninth intercostal space. There were no special arrangements associated with the port settings compared to the settings during a normal esophagectomy. A 30° 10-mm videoscope was used and artificial pneumothorax using CO_2_ insufflation was maintained at 6 mmHg. The operative field visibility was not inferior to that of a usual esophagectomy in the upper and middle mediastinum; however, the lower mediastinum was narrow because of PE (Fig. 2a, d). The operation was difficult, especially lymph node dissection along the left pleura. An additional movie file shows this in more detail [see Additional file 1]. After esophagectomy, a gastric conduit was created laparoscopically, pulled up through the posterior mediastinal route, and the cervical esophagogastric anastomosis was then performed. The operating time was 457 min, and the estimated blood loss was 121 g. Pathological staging indicated stage IB (pT2, pN0, cM0). The postoperative course was uneventful, and the patient was discharged on postoperative day 13. The patient survived without recurrence for over 24 months postoperatively.

### Patient 2

**Fig. 3:**
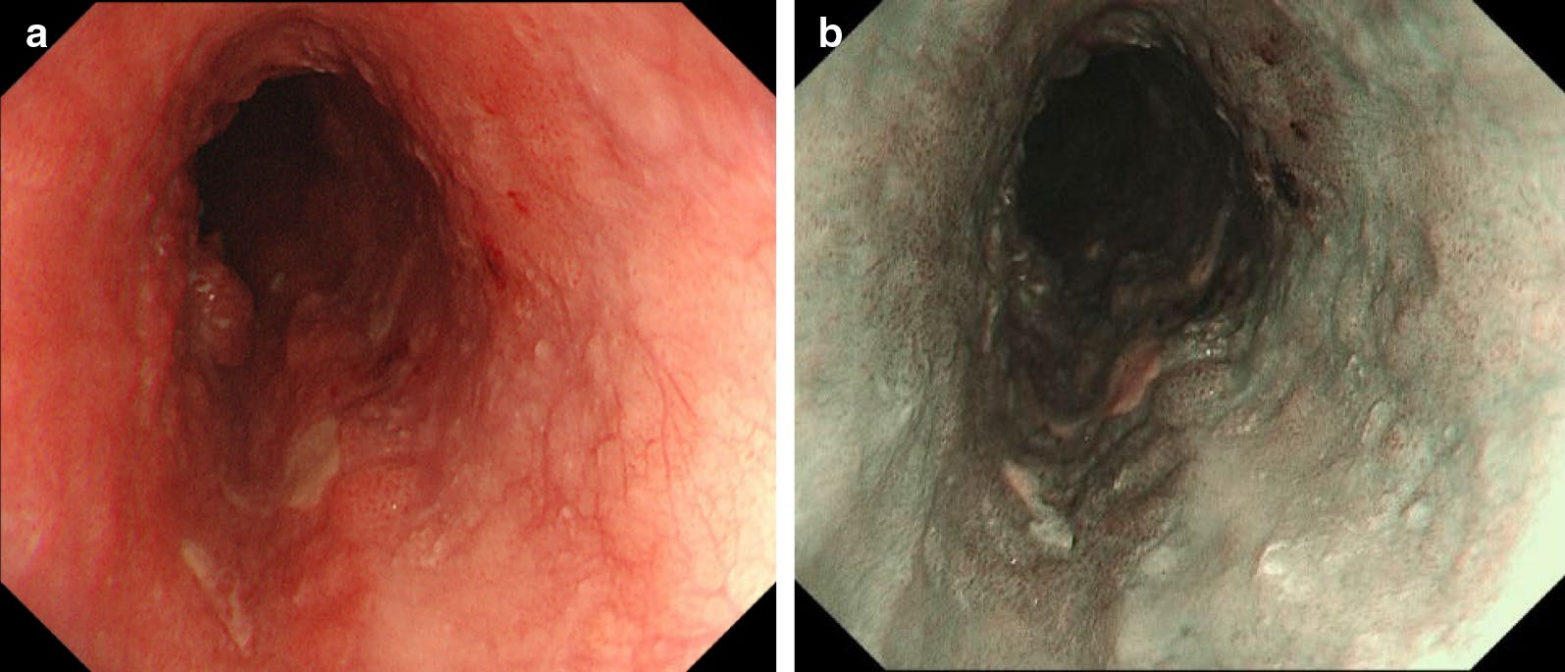
Patient 2. **a** Endoscopic findings. The widespread flat lesion in the middle esophagus was detected at 35–40 cm from the upper incisors, and suspected submucosal invasion. **b** Narrow band imaging

**Fig. 4:**
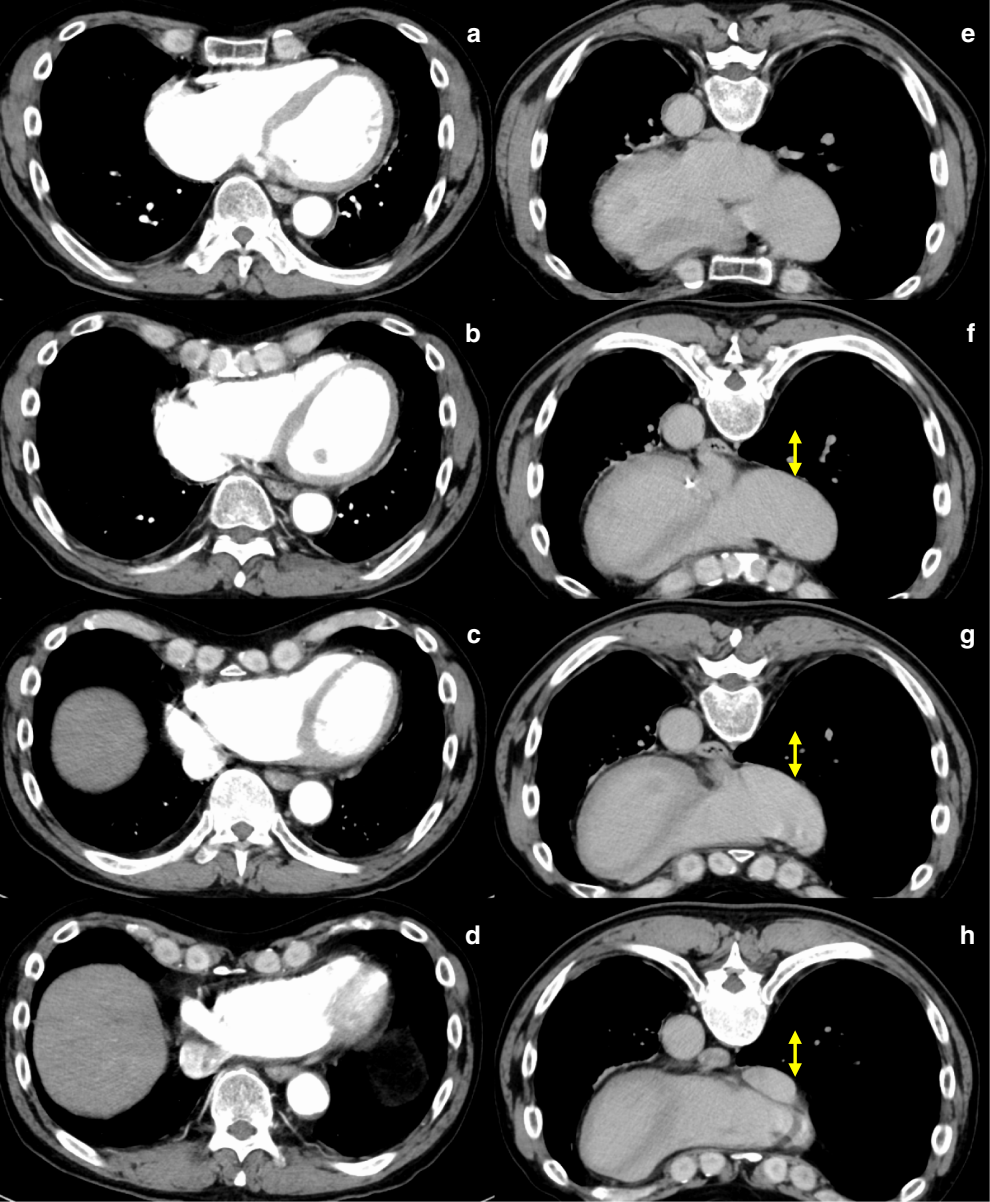
Patient 2. **a**–**d** Contrast-enhanced CT in the supine position showing the pectus excavatum. The Haller index score was 4.3. **e**–**h** CT image in the prone position revealed dilated mediastinum (arrow) compared to **a**–**d**. CT slice level was matched based on the sternal bone

**Fig. 5:**
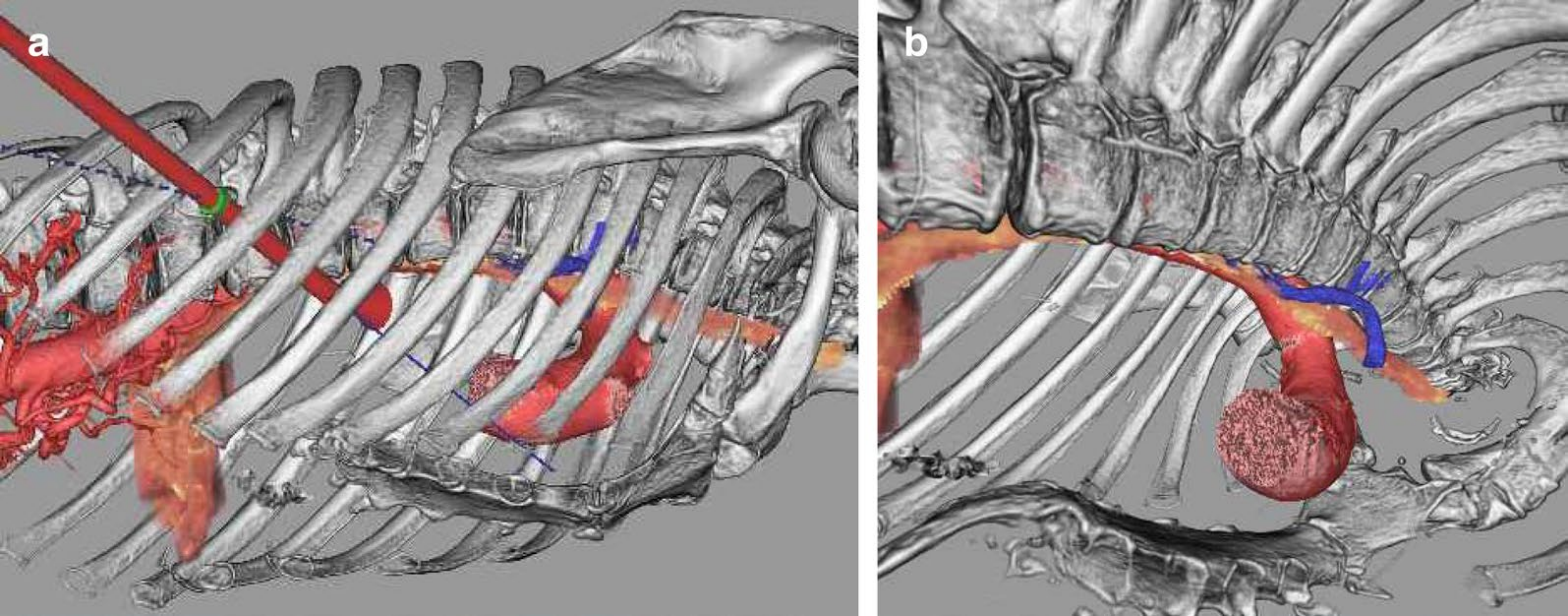
**a** 3D-CT image created by contrast-enhanced CT images in the supine position. **b** 3D-CT imaging revealed difficulty in ensuring the surgical field

**Fig. 6:**
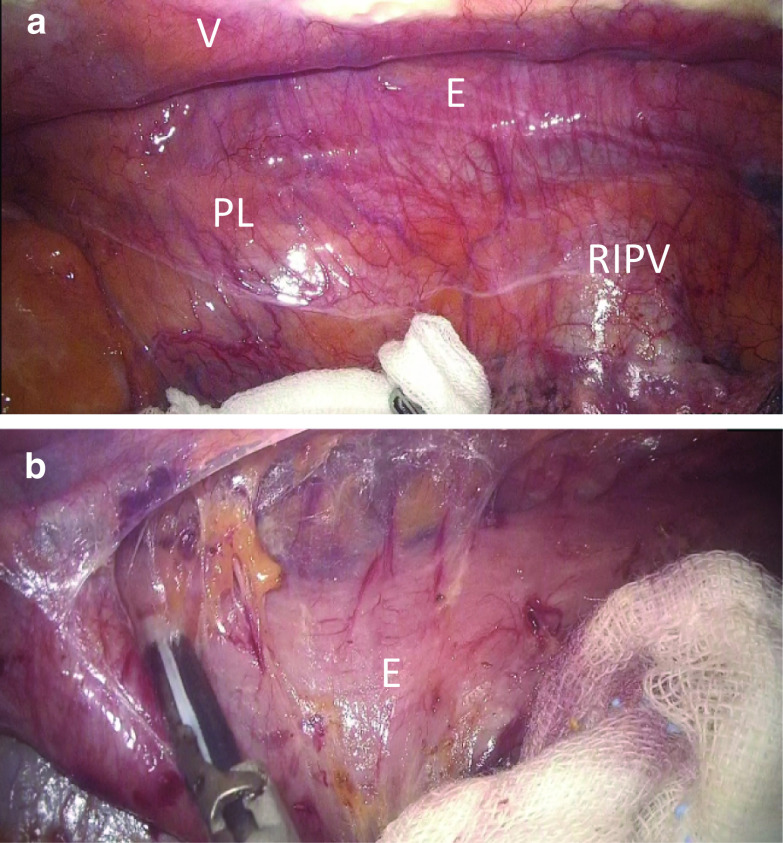
Thoracoscopic intraoperative findings of Patient 2. **a** Esophagus can be seen from a top-down view. **b** Dissection of the dorsal side of esophagus. The operative field visibility was better than that in patient 1**.** *V* Vertebra, *PL* pulmonary ligament, *RIPV* right inferior pulmonary vein, *E* esophagus

A 67-year-old Asian man was diagnosed with esophageal cancer during an annual routine medical checkup. His medical history included anti-phospholipid antibody syndrome with regular use of 2.5 mg of warfarin. Upper gastrointestinal endoscopy showed a widespread flat lesion in the middle esophagus, 35–40 cm from the upper incisors (Fig. 3a, b), and biopsy indicated moderately differentiated squamous cell carcinoma. Contrast-enhanced CT could not detect the main tumor, but revealed PE with a Haller index of 4.3 (Fig. 4a–d). A CT scan in the prone position was also performed (Fig. 4e–h). However, the patient had no clinical symptoms associated with PE. The preoperative diagnosis was stage I squamous cell carcinoma of the esophagus (cT1b, cN0, cM0). Using Digital Imaging and Communication in Medicine (DICOM) data in the spine position, three-dimensional (3D) images were created using volume analysis software, SYNAPSE VINCENT™ (Fujifilm Medical, Tokyo, Japan) for preoperative simulation (Fig. 5a, b). Using these, we assessed that the mediastinum of this patient was relatively wider than that of patient 1; therefore, only esophagectomy was performed. Radical thoracoscopic esophagectomy, with two-field lymph node dissection, was performed with the patient in the prone position. The operative field visibility was better than that obtained in patient 1 (Fig. 6a, b). The operating time was 413 min, and the estimated blood loss was 34 g. The pathological staging indicated stage IB (pT1b, pN0, cM0). The postoperative course was uneventful, and the patient was discharged on postoperative day 17. The patient was in good condition at the 18-month follow-up visit.

## Discussion and conclusion

In radical esophagectomy, especially in minimally invasive esophagectomy (MIE), the narrow mediastinum, a typical feature of PE, can increase the difficulty of surgery [8]. The Haller index score is widely used to evaluate the severity of PE; the normal range is from 2.5 to 2.7, whereas a value of 3.25 or higher is indicative of a need for surgery. The Nuss procedure [9], a treatment method for PE in which a metal intrathoracic bar is placed behind the sternum under a thoracoscope, is currently widely used in adults and has low complication rates and short hospital stays. However, a surgery is required to remove the metal bar 2–3 years later.

To the best of our knowledge, there are only two previous reports of patients with PE who have undergone thoracoscopic esophagectomy (Table 1). However, both cases were performed with the patients in the left lateral decubitus position. While Sato et al. reported the usefulness of video-assisted thoracoscopic surgery in a case of a patient with Haller index of 4.83 [3], Hatoyama et al. reported a case of a patient with a severely narrow mediastinum (Haller index, 9.9) and recommended a simultaneous PE repair by Nuss procedure [4].

**Table 1:** Reported cases of patients with pectus excavatum who have undergone thoracoscopic esophagectomy

Year	Author	Age	Gender	Haller index	Position	Approach	Reconstruction	Route	Sternal repair	Hospital stay	Complication	Prognosis
2015	Sato et al. [3]	77	Male	4.83	Left lateral	Thoracoscopic	Gastric conduit	PMR	None	POD 116	Brain infarction, leakage	ND
2018	Hatoyama et al. [4]	59	Male	9.9	Left lateral	Thoracoscopic	Gastric conduit	PMR	Nuss	ND	None	2 years alive
	Our case 1	64	Male	8.5	Prone	Thoracoscopic	Gastric conduit	PMR	None	POD 13	None	24 months alive
	Our case 2	67	Male	4.3	Prone	Thoracoscopic	Gastric conduit	PMR	None	POD 17	None	18 months alive

*PMR* posterior mediastinal route, *POD* postoperative day, *ND* not described

MIE in the prone position is generally considered to provide a better surgical view of the mid-lower mediastinum than that in the left lateral position. In our study, the patients had no clinical symptoms associated with PE; therefore, we decided not to perform PE repair simultaneously. The most important factor that contributed to the difficulty of the surgery, especially in Case 1, was the relatively high Haller index score, because it was difficult to maintain a good surgical view. Furthermore, patients with bulky tumors or apparent metastatic lymph nodes with a narrow mediastinum are more difficult to operate and may require simultaneous PE repair. 3D-CT evaluation and a CT in the prone position before surgery, as used in Case 2, can help predict if the mediastinal field of view could be secured; the surgery can then be performed with relative ease.

During perioperative management, MIE in the prone position is associated with some disadvantages during emergent thoracotomy. If an accident such as uncontrollable intraoperative bleeding occurs, the left lateral decubitus and prone hybrid position enables us to immediately convert from thoracoscopic to open surgery [10]. Regarding reconstruction, it is necessary to use a narrowed (3–3.5 cm) gastric conduit and to raise it slightly to the right of the midline in order to avoid cardiopulmonary compression during posterior mediastinal route reconstruction. In cases of simultaneous PE repair, pain control is important as a postoperative management that should be noted. In addition, infectious complications after the Nuss procedure are potentially devastating [11]. From our experience, patients who are asymptomatic preoperatively do not have many problems after surgery. However, the posterior mediastinal route reconstruction may cause tachyarrhythmia due to retraction of the left atrium by the reconstructed organ.

In previous large-scale reports of cases treated with the Nuss procedure, the mean Haller index was 5.15 ± 2.32 (mean ± SD) [9]. Therefore, based on previous case reports [3, 4] and our experience, in cases with a Haller index is 5 or higher, simultaneous PE repair may be indicated. However, performing MIE in the prone position cannot be recommended after PE repair because of sternal instability. In advanced cases of PE, as reported by Hatoyama et al., left lateral decubitus esophagectomy, which allows simultaneous PE repair, may be preferred [4]. However, in cases with a relatively low Haller index score (< 5), specifically in cases with large mediastinal space as confirmed by preoperative CT in the prone position (as in Case 2), a good surgical view can be secured in the prone position without simultaneous PE repair.

A trans-mediastinal or trans-hiatal approach [12] can be considered in similar cases. However, regarding reconstruction, when using any kind of approach to esophagectomies, surgeons should note that the anterior thoracic and posterior sternal routes are considered unfavorable with PE repair because of technical difficulty. To avoid injuring the gastric conduit, a posterior mediastinal route must be selected. The posterior sternal route may also be an option for patients with relatively mild (the Haller index < 5) and asymptomatic PE who do not need a simultaneous repair.

In conclusion, radical thoracoscopic esophagectomy in the prone position can be explored as a surgical option if PE repair is not required. The indications for simultaneous PE repair are still controversial. Further case accumulations are required. Furthermore, preoperative imaging in the prone position was found to be a help while determining the appropriate surgical procedure.

## Supplementary Information

**Additional file 1.** Summary video of the surgery in Patient 1**. **Thoracoscopic video of mid-lower mediastinal area in the prone position.

## Data Availability

Data sharing is not applicable to this article, since datasets were neither generated nor analyzed for the case report.

## Abbreviations

PE: Pectus excavatum
CT: Computed tomography
UICC: Union for International Cancer Control
DICOM: Digital Imaging and Communication in Medicine
MIE: Minimally invasive esophagectomy
